# Advantages of ultrasound imaging for the early diagnosis of psoriatic arthritis in patients with moderate to severe psoriasis

**DOI:** 10.1016/j.heliyon.2024.e34136

**Published:** 2024-07-04

**Authors:** Rongfen Chen, Xiaoyuan Zhong, Dawei Huang, Zitong Chen, Yingyuan Yu, Jiajing Lu, Qiao Wang, Luyang Kong, Xuemei Yi, Yujing Zhao, Yangfeng Ding, Lehang Guo, Yuling Shi

**Affiliations:** a Department of Dermatology, Shanghai Skin Disease Hospital, Tongji University School of Medicine, Shanghai, 200443, China; b Institute of Psoriasis, Tongji University School of Medicine, Shanghai, 200443, China; c Department of Medical Ultrasound, Shanghai Tenth People's Hospital, Shanghai, China; d Department of Medical Imaging, Shanghai Skin Disease Hospital, Tongji University School of Medicine, Shanghai, China

**Keywords:** Psoriasis, Psoriatic arthritis, Ultrasound, Subclinical stage, Timely diagnosis

## Abstract

**Background:**

Psoriatic arthritis (PsA) is an immune-mediated form of chronic inflammatory arthritis associated with psoriasis (PsO). It constitutes a significant comorbidity of PsO and is distinguished by the presence of widespread musculoskeletal inflammation.

**Objective:**

The aim of this study is to precisely detect asymptomatic PsA using ultrasound (US) examinations and to distinguish between various stages of PsO.

**Methods:**

All patients with moderate-to-severe PsO, who consented to undergo musculoskeletal US examinations during their hospitalization between September 2020 and January 2022, were enrolled in the study. We compared patients' demographic characteristics, comorbidities, disease duration, relevant laboratory parameters, and musculoskeletal US findings.

**Results:**

A total of 547 patients with PsO were included in the study, and 114 of them received a diagnosis of PsA. Furthermore, 16.45 % of patients with moderate to severe PsO displayed subclinical PsA. We observed a significantly higher frequency of abnormal US findings in patients with PsA compared to those without PsA, with a sensitivity of 95.61 % and a specificity of 79.22 %. Additionally, the incidence of enthesitis and synovitis varied significantly between PsA and non-PsA patients, and they were identified as independent variables predicting the presence of PsA. Furthermore, the interphalangeal joint, knee joint, and calcaneal tendon were the most frequently affected areas in PsA, as indicated by the observed US changes.

**Conclusion:**

Ultrasound examination proves to be a valuable tool for detecting subclinical PsA, facilitating early screening of the condition. Particular attention should be directed towards changes in the interphalangeal joint, knee joint, and calcaneal tendon when reviewing ultrasound images of asymptomatic patients.

## 1 Introduction

Psoriatic arthritis (PsA) is an immune-mediated form of chronic inflammatory arthritis associated with psoriasis (PsO). It represents a significant comorbidity of PsO and is characterized by widespread musculoskeletal inflammation [1]. PsA has a profound impact on patients' quality of life, with joint deformities present in 40 % of PsA patients and severe disruption of daily activities in 34 % of cases [2,3]. A systematic review and meta-analysis revealed that the overall prevalence of PsA in patients with PsO was 19.7 %, and in individuals with moderate-to-severe PsO, it reached as high as 24.6 % [4]. However, in China, the reported incidence of PsA among patients with psoriasis ranges from 6 % to 13 % [5].

Recent developments have proposed a five-stage characterization for the transition from PsO to PsA [[6], [7], [8]], encompassing PsO, preclinical PsA, subclinical PsA, prodromal PsA, and clinical PsA. Subclinical PsA is defined as PsO with evidence of silent synovio-entheseal inflammation on imaging but without significant arthritis symptoms. In contrast, patients in the clinical PsA stage exhibit inflammatory articular disease and meet the CASPAR diagnostic criteria for PsA. Currently, clinical treatment of PsA primarily concentrates on the clinical PsA stage. Nevertheless, during this stage, some PsA patients may have already developed severe joint deformities, and there is limited information available regarding the outcomes of clinical interventions [2,3]. Therefore, early diagnosis and timely intervention for PsA patients before they progress to the clinical PsA stage are of paramount importance.

Early diagnosis of PsA is challenging because the joint and skin severity of PsA are not consistent. Consequently, patients often experience significant adverse impacts on their quality of life, economic circumstances, family dynamics, and psychological well-being [9]. Presently, the diagnosis of PsA heavily relies on the CASPAR diagnostic criteria established in 2006 [10]. To meet the CASPAR criteria, a patient must exhibit inflammatory articular disease involving joints, the spine, or entheses. Additionally, various questionnaires have been developed for screening and identifying individuals with PsA, including the Psoriasis and Arthritis Screening Questionnaire (PASQ) [11], the Psoriasis Epidemiology Screening Tool (PEST) [12], the Toronto Psoriasis-Arthritis Screen (ToPAS) [13], the Simple Psoriatic Arthritis Screening (SiPAS) questionnaire [14], and Early Psoriasis-Arthritis Screening (EARP) [11]. However, these tools are primarily effective in diagnosing PsA in patients with subjective symptoms, potentially overlooking those who are temporarily asymptomatic. Given the diagnostic challenges of subclinical PsA, dermatologists may need to employ adjunctive tests to conduct a more comprehensive clinical assessment of patients with early or subclinical PsA. Auxiliary examinations for PsA primarily encompass imaging techniques such as x-rays, ultrasonography, and magnetic resonance imaging (MRI).

The attachment point serves as the insertion site for tendons and ligaments on the bone's surface. Inflammation of attachment point often represents an early prominent characteristic of PsA [15]. Clinical physical examinations have revealed that approximately 30 % of PsA patients experience attachment inflammation [16] Synovitis, a secondary inflammation, intensifies the inflammation at the attachment points in PsA and results in structural damage to the joint synovium [17]. Previous studies have indicated that synovitis primarily manifests in the later stages of inflammation. Asymptomatic synovitis exhibits a higher prevalence in PsA, making its identification challenging [[18], [19], [20]]. Early changes in articular soft tissue are difficult to detect via X-rays, particularly when diagnosing enthesitis in early PsA [21,22]. MRI can reveal inflammation-related features of arthritis and is, therefore, useful for the early diagnosis of axial PsA [23]. However, MRI examinations necessitate the use of gadolinium-containing contrast agents, which can pose potential toxicity risks, and the procedure is time-consuming and expensive [24]. Unlike tissue inflammation, X-rays are advantageous for detecting structural lesions such as erosion. Ultrasound (US) imaging offers several advantages over X-rays and MRI, as it can identify early PsA manifestations in the skin, nails, joints, and entheses in PsO patients [25]. It is a relatively more sensitive and convenient modality for detecting changes in the joint, tendon, and enthesis compared with physical examination and other imaging methods [26,27]. Furthermore, US imaging is highly portable, easily accessible, and cost-effective [28].

Nevertheless, when the CASPAR criteria for PsA were introduced in 2006, there was limited literature addressing the potential value of US imaging. Consequently, abnormal US features were not integrated into this set of criteria. Therefore, this study explores whether abnormal US features can assist clinicians in evaluating and staging asymptomatic PsA patients.

## 2 Materials and methods

### 2.1 Study design

This cross-sectional study was conducted at the Dermatology Hospital from September 2020 to January 2022. The Ethics Committee of the Shanghai Skin Disease Hospital (#2020-36) thoroughly reviewed this prospective cohort study. All participants provided informed consent, and their privacy and confidentiality were carefully safeguarded throughout the study.

### 2.2 Participants

All participants in the study were recruited from outpatient clinics and wards at Shanghai Skin Disease Hospital. Inclusion criteria were as follows: 1. Adults diagnosed with moderate-to-severe plaque psoriasis[29]; 2. Patients undergoing joint US examinations and CASPAR assessment; 3. Venous blood samples were collected for routine blood tests and rheumatoid factor (RF) tests. Main exclusion criteria included: 1. Diagnoses of other forms of arthritis, such as rheumatoid arthritis, osteoarthritis, gouty arthritis, ankylosing spondylitis, or similar conditions; 2. Current pregnancy; 3. History of trauma or extensive manual labor prior to the US examination.

### 2.3 Study procedure

When patients were recruited for the study, an independent study coordinator recorded their general information, including age, gender, body mass index (BMI), smoking history, and drinking history. This was followed by an independent evaluation of psoriasis and joint assessment by a dermatologist and a rheumatologist. The evaluation of psoriasis included an assessment of the Psoriasis Area and Severity Index (PASI), duration of previous psoriasis, family history of psoriasis, and nails evaluation. The joint assessment included an evaluation of joint symptom，finger/toe inflammation and CASPAR assessment. In light of the radiation associated with X-rays, patients may be hesitant to undergo this imaging technique. Therefore, patients were initially assessed using the CASPAR criteria without X-rays, and only ultrasound tests were conducted if the patient scores at least 3. However, if a patient cannot be diagnosed with PsA using the CASPAR criteria without X-rays, they were required to undergo both ultrasound and X-ray evaluations. Subsequently, all patients underwent US and/or X-ray examinations for further imaging evaluation. 5 ml of fasting blood was collected from a vein for laboratory examination, containing erythrocyte sedimentation rate (ESR), C-reactive protein (CRP), RF, uric acid (UA) levels, neutrophil count, lymphocyte count, and platelet count.

### 2.4 Imaging evaluation of the joint

Due to the variable location of PsA and the variety of clinical types, we performed US and/or X-ray examinations on the joints of all patients. However, considering the limitations of ultrasonic detection for axial joints, the range of joints we detected only included those in the hands, knees, and feet, covering 60 joints, 38 tendons, and 40 entheses. Each US and X-ray scan was performed under pre-set musculoskeletal parameters by two radiologists, who had 5 and 7 years of experience, respectively, in musculoskeletal US and X-ray imaging and came to an agreement on the standardized US and X-ray definitions. Before scanning, the two radiologists had received strict training and achieved an almost perfect interoperator agreement (Kappa value > 0.80). All images were stored for further analysis. Two other radiologists blinded to both the clinical assessments of psoriasis participants reviewed the US and X-ray images independently. Considering the limited use of joint ultrasound in evaluating PsA, the descriptive definitions for US are listed in Table 1[30].

**Table 1:** “US defnitions of lesions”.

Ultrasonographic lesion	Definition
Enthesitis	Hypoechoic and/or thickened insertion of the enthesis close to the bone (within 2 mm from the bony cortex) which exhibits Doppler signal if active and that may show erosions, enthesophytes/calcifcations as sign of structural damage.
Synovitis	Presence of a hypoechoic synovial hypertrophy regardless of the presence of effusion or any grade of Doppler signal.
Tenosynovitis	Tenosynovial hypertrophy is defined as presence of abnormal hypoechoic (relative to tendon fibers) tissue within the synovial sheath that is not displaceable and poorly compressible, and seen in two perpendicular planes; it may exhibit Doppler signals.
Joint effusion	Erosions were defined as intra-articular discontinuity of the bone surface that is visible in two perpendicular planes.
New bone formation	New bone formation is defined as hyperechoic bony prominence distant from the bone margin.

### 2.5 Diagnosis of PsA

Dermatologists and rheumatologists used the CASPAR criteria to diagnose patients with PsA. Diagnostic assessments were based on the patient's joint symptoms, psoriasis assessment, psoriatic nail changes, finger/toe inflammation, rheumatoid factor (RF), and X-ray assessments. Patients with a score of 3 or higher were diagnosed with PsA.

### 2.6 Statistical analysis

Statistical analyses were conducted using the Statistical Package for the Social Sciences version 20.0 (SPSS Inc., Chicago, IL, USA). Continuous quantitative data, which were normally distributed, were presented as mean ± standard deviation and analyzed using the student's t-test. Continuous quantitative data that did not conform to a normal distribution were expressed as the median and interquartile range and analyzed using the Mann-Whitney *U* test. Qualitative data were reported as the number of cases and corresponding percentages and subjected to analysis using the chi-square test. The Pearson correlation coefficient was used to assess the degree of correlation between variables. Logistic binary regression was employed to investigate the factors influencing the occurrence of subclinical joint damage in patients with psoriasis. A significance level of P < 0.05 was considered indicative of statistically significant differences.

## 3 Results

### 3.1 Evaluation of PsA

**Fig. 1:**
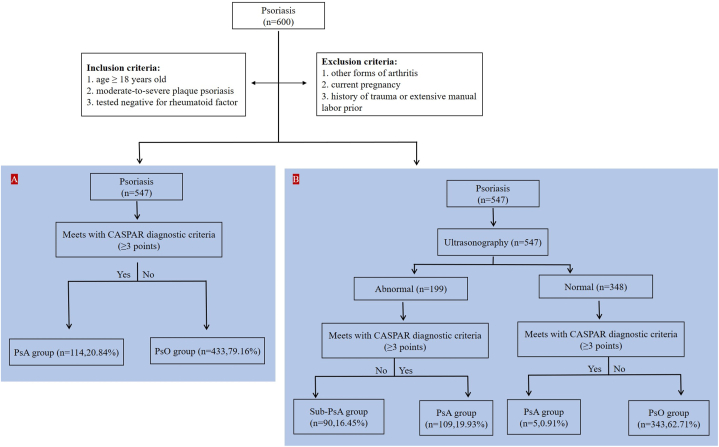
“Flow diagram of patient screening with two diagnostic approaches.” (A) With the first approach, based on the CASPAR diagnostic criteria, patients were divided into the PsA and PsO group. (B) With the second approach, patients were divided into the subclinical PsA (Sub-PsA) group, the PsO group, and the PsA group.

A total of 600 patients were recruited, of whom 547 patients with psoriasis completed the study, meeting the atretic criteria. In total, 114 patients (20.84 %) were diagnosed with PsA using the CASPAR diagnostic criteria, while excluding other possible forms of arthritis. However, this method only allows for classification of patients into PsA and non-PsA categories. Therefore, we attempted to assess PsA in patients using ultrasound examination. In this approach, all patients underwent joint ultrasound as a first step, leading to the identification of ultrasound abnormalities in 199 patients. Among these, 109 were diagnosed with PsA according to CASPAR diagnostic criteria. Additionally, 90 patients (16.45 %) with psoriasis did not exhibit any joint symptoms, leading to their classification as having subclinical PsA based on previous literature [[6], [7], [8]] (Fig. 1). Interestingly, five patients presented with joint symptoms, primarily experiencing joint pain and swelling. However, no abnormalities were detected on US and X-ray. Nevertheless, these five patients still met the criteria for diagnosis of PsA according to CASPAR criteria.

### 3.2 Differences in characteristics between the PsA and Non-PsA groups

We investigated in detail differences in the clinical characteristics of patients in the PsA and non-PsA groups (Table 2). Among the various clinical characteristics that were examined, there were significant differences in age, gender, incidence of abnormal US findings, ESR, and CRP. The group of patients with PsA contained more individuals over the age of 40, fewer men, and more individuals who smoke and drink. Additionally, patients with PsA exhibited higher ESR and CRP levels, and more abnormalities on ultrasound tests. The incidence of abnormal US manifestations was significantly higher in patients with PsA, with a sensitivity of 95.61 % and a specificity of 79.22 %.

**Table 2:** “Demographic and clinical characteristics of the patients (N = 547).”

	Non-PsA (n = 433)	PsA (n = 114)	*P*
Age, median (IQR), years	59.00 (26.00)	59.00 (18.50)	0.405
≥40 years, n（%）	330 (76.21)	97 (85.09)	0.039
Gender, male, n (%)	352 (81.29)	82 (71.93)	0.018
BMI, mean ± SD	24.69 ± 3.86	24.94 ± 3.67	0.557
BMI ≥24, n (%)	230 (53.12)	69 (60.52)	0.196
Family history of psoriasis, n (%)	56 (12.93)	19 (16.67)	0.218
Current smoker, n (%)	148 (34.18)	53 (46.49)	0.019
Current drinker, n (%)	70 (16.17)	36 (31.58)	<0.001
Nail dystrophy, n (%)	272 (62.82)	75 (65.79)	0.825
PASI, median (IQR)	15.50 (10.00)	14.95 (9.88)	0.191
ESR, median (IQR)	10.00 (10.00)	15.00 (18.00)	<0.001
CRP, median (IQR)	2.80 (7.10)	5.20 (20.35)	0.001
US findings			
Abnormal, n (%)	90 (20.78)	109 (95.61)	<0.001
Normal, n (%)	343 (79.22)	5 (4.39)	

ESR: Erythrocyte sedimentation rate, CRP: C-reactive protein, Non-PsA: Sub-PsA and PsO group, PsA: psoriatic arthritis, BMI: body mass index, PASI: psoriasis area and severity index.

### 3.3 Differences in characteristics between patients with normal and abnormal US findings

Table 3 summarizes the clinical features of patients with abnormal and normal US findings. There were 199 and 348 patients with abnormal and normal ultrasound findings, respectively. In the group with abnormal US findings, a significantly higher number of patients were ≥40 years than in the group with abnormal US findings. In addition, patients with abnormal US findings had significantly higher ESR, CRP, and BMI than those with normal US findings.

**Table 3:** “Characteristics of patients with and without abnormal US findings.”

	Normal US findings (n = 348)	Abnormal US findings (n = 199)	*P*
Age, median (IQR) years	57.00 (27.00)	60.00 (19.25)	0.021
Gender, male, n (%)	284 (81.61)	150 (75.38)	0.099
BMI, median (IQR)	24.10 (4.77)	24.22 (4.87)	0.033
Family history of psoriasis, n (%)	43 (12.36)	32 (16.08)	0.249
Current smoker, n (%)	116 (33.33)	83 (41.71)	0.066
Current drinker, n (%)	53 (15.30)	52 (26.13)	0.002
Nail dystrophy, n (%)	222 (63.79)	120 (60.30)	0.304
PASI, median (IQR)	15.20 (9.00)	15.00 (1.50)	0.652
ESR, median (IQR)	2.70 (6.45)	4.40 (15.25)	<0.001
CRP, median (IQR)	10.00 (11.00)	13.00 (15.00)	0.013

ESR: Erythrocyte sedimentation rate, CRP: C-reactive protein, PsA: psoriatic arthritis; BMI: body mass index, PASI: psoriasis area and severity index.

### 3.4 Univariate analysis of abnormal US findings as indicators of PsA

In order to determine whether the US abnormalities identified as being associated with PsA were indeed significant indicators of the condition, univariate analysis of the abnormal US findings was performed. The results showed that enthesitis and synovitis were significant predictors of PsA (Table 4).

**Table 4:** “Univariate analysis of US abnormalities as indicators of PsA.”

Characteristics	Univariate analysis		Multivariate analysis	
	Odds Ratio (95 % CI)	P value	Odds Ratio (95 % CI)	P value
Age	1.006 (0.992–1.019)	0.428		
Gender				
Male	Reference		Reference	
Female	1.794 (1.111–2.897)	0.017	2.662 (1.453–4.879)	0.002
Family history of psoriasis				
No	Reference			
Yes	1.372 (0.777–2.425)	0.276		
BMI	1.002 (0.950–1.058)	0.932		
Current smoker				
No	Reference		Reference	
Yes	1.685 (1.100–2.583)	0.017	1.274 (0.670–2.421)	0.460
Current drinker				
No	Reference		Reference	
Yes	2.448 (1.517–3.951)	<0.001	2.218 (1.088–4.522)	0.028
Nail dystrophy				
No	Reference			
Yes	1.061 (0.684–1.645)	0.793		
Enthesitis				
No	Reference		Reference	
Yes	5.891 (3.702–9.373)	<0.001	3.471 (1.934–6.229)	<0.001
Synovitis				
No	Reference		Reference	
Yes	33.170 (9.682–113.639)	<0.001	27.326 (7.156–104.355)	<0.001
Tenosynovitis				
No	Reference		Reference	
Yes	12.232 (4.301–34.785)	<0.001	5.867 (1.603–21.473)	0.008
Joint effusion				
No	Reference		Reference	
Yes	3.873 (1.714–8.752)	0.001	1.301 (0.464–3.650)	0.617
New bone formation				
No	Reference		Reference	
Yes	5.603 (3.205–9.797)	<0.001	3.144 (1.558–6.346)	0.001
PASI	0.983 (0.956–1.012)	0.249		

### 3.5 Differences in the frequency of abnormal US findings between the PsA and sub-PsA groups

**Fig. 2:**
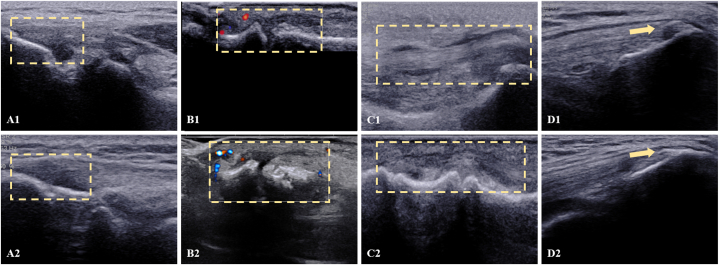
“Difference in US abnormalities between the PsA and Sub-PsA groups.” (A) Fig. 2 PsA (A1-D1) and Subclinical PsA (A2-D2) ultrasound abnormalities. A1, A2: Enthesitis (dotted boxes), manifested as hypoechoic thickened insertion of the enthesis close to the bone; B1, B2: Synovitis (dotted lines), manifested as hypoechoic synovial hypertrophy; C1, C2:Tenosynovitis (dotted boxes), manifested as abnormal hypoechoic tendon sheath widening; and D1, D2: New bone formation (arrows), manifested as hyperechoic bony prominence distant from the bone margin.

Based on US test results, we classified patients with abnormal US test into PsA and Sub-PsA, and compared the US characteristics between them (Table 5). The frequency of synovitis in patients with PsA was significantly higher than that in patients without PsA. On the contrary, the frequency of enthesitis was significantly higher in patients with subclinical PsA than those with PsA. US images depicting these features in the PsA and Sub-PsA groups are shown in Fig. 2 (A1-D1, A2-D2).

**Table 5:** “Frequency of abnormal US findings between the PsA and Sub-PsA groups.”

	Sub-PsA (n = 90)	PsA (n = 109)	*P*
Enthesitis, n (%)	61 (67.78)	54 (49.54)	0.014
Synovitis, n (%)	4 (4.44)	29 (26.61)	<0.001
Tenosynovitis, n (%)	5 (5.56)	14 (12.84)	0.094
Joint effusion, n (%)	12 (13.33)	12 (11.01)	0.666
New bone formation, n (%)	31 (33.33)	32 (29.36)	0.449

### 3.6 Frequency of abnormal US findings in different regions according to PsA stage

**Fig. 3:**
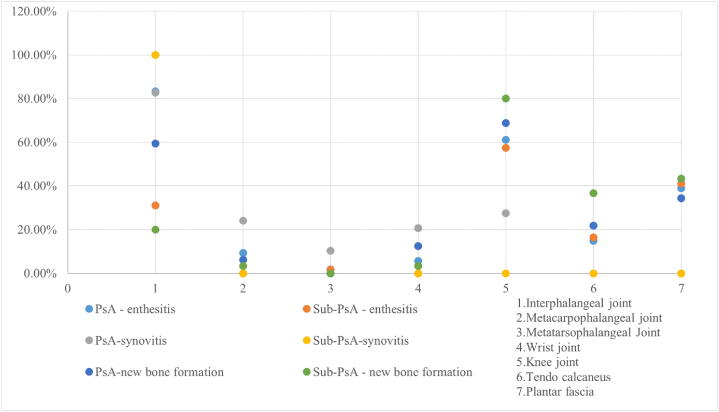
“Frequency of new bone formation, enthesitis, and synovitis at various sites in the Sub-PsA and PsA groups.”

Although ultraso/nography can be used to detect PsA with high sensitivity, it is crucial to determine which joints the examination should focus on in patients without subjective symptoms. New bone formation is one of the diagnostic criteria according to the CASPAR guidelines [6,10], and enthesitis and synovitis were identified as significant predictors of PsA in the present study. Therefore, we summarize in Fig. 3 the sites of new bone formation, enthesitis, and synovitis in the Sub-PsA and PsA groups. The frequency of new bone formation, enthesitis, and synovitis was higher in the interphalangeal joint, knee joint, plantar fascia, and tendon calcaneus than in the other regions included.

## 4 Discussion

This study underscores the merits of employing ultrasound (US) imaging to diagnose Psoriatic Arthritis (PsA) in the Chinese population across various joint sites. Our findings revealed a noteworthy prevalence of PsA among patients with moderate-to-severe Psoriasis (PsO), reaching as high as 20.84 %, a statistic considerably exceeding prevalence rates reported in other studies involving the Chinese population [4,5]. This disparity may be attributed to the composition of our study population, predominantly comprising individuals with moderate-to-severe PsO. Moreover, our utilization of joint US imaging unveiled that 16.45 % of all patients presenting with moderate-to-severe PsO exhibited subclinical PsA. Notably, as of now, there are no established guidelines for diagnosing subclinical PsA based on US findings, rendering these discoveries quite valuable.

Furthermore, our study disclosed that the frequency of abnormal US findings was significantly elevated in patients with PsA, resulting in a remarkable sensitivity of 95.61 % and a specificity of 79.22 %.

When joint inflammation occurs, the entheses often become the initial site affected [31]. Our study revealed a substantial increase in the incidence of enthesitis among patients with both subclinical PsA and clinical PsA. Therefore, in general, enthesitis in PsA may represent an early manifestation of joint involvement and therefore has a higher incidence in patients with subclinical PsA. As the disease progresses, joint involvement becomes more pronounced, and the types of damage increase. Enthesitis manifests as tendon and ligament thickening, muscle fiber irregularities, and the enhancement of blood flow signals in US images [32]. However, several other studies have noted that enthesial thickening can also occur in otherwise healthy individuals. For example, Di Matteo et al. [33] found that 28.0 % (23/82) of healthy subjects had entheseal thickening. This condition has been described as a physiological state resulting from prolonged mechanical pressure and higher body mass index (BMI) [34]. However, it has been reported that enthesitis is one of the characteristic manifestations of PsA [31], and our study indicated that as many as 67.78 % of patients with subclinical PsO may already exhibit enthesitis. In light of its prevalence among individuals without PsA, the observation of physiological thickening at attachment points on US images should be approached with caution.

In our study, a significantly higher percentage of patients with clinical PsA than with subclinical PsA had synovitis. In fact, based on our data, synovitis was rarely observed in patients with subclinical PsA. A previous study reported that synovitis is more common in chronic forms of PsA [20].This observation may elucidate the association of synovitis primarily with the clinical PsA stage, rather than the subclinical phase.

Traditionally, bone destruction is considered as a severe late-stage manifestation of PsA [35]. However, in the present study, new bone formation and joint effusion were also detected in the subclinical PsA stage. It is well known that bone destruction is related to micro-injuries, immune activation, and microvascular proliferation. Accordingly, significant vascularization has been observed even in the subclinical PsA stage [34]. These findings suggest the potential presence of abnormal immune activation in PsA and underscore the pivotal role of inflammation during the subclinical phase.

Subjective joint symptoms in patients with PsA in the clinical stage often involve the interphalangeal joints, metacarpophalangeal joints, metatarsophalangeal joints, and other small joints of the hands and feet [36,37]. However, the present study found that interphalangeal joint involvement can be observed with US imaging even during the subclinical stage. Therefore, US scanning to detect the potential involvement of interphalangeal joints is recommended in the early stage of the disease. The present study also found that significant involvement of the knee joint, plantar fascia, and tendon calcaneus in the subclinical PsA stage. Therefore, imaging of the knee joint, plantar fascia, and tendon calcaneus is also recommended in PsO patients without articular symptoms.

Our research has some limitations. First, it has a cross-sectional design, so long-term follow-up of patients will be required in the future to confirm the findings. Secondly, US imaging was not compared with other imaging modalities such as MRI. Despite this, US scanning still has enormous advantages from the screening perspective. Third, there were some limitations in the assessment of the central axis joint, so further research on US abnormalities in this area is required. Furthermore, it's important to note that our study primarily concentrates on a specific Chinese population group, and as such, the generalizability of the results to other populations may be limited.

## 5 Conclusion

In summary, our study unveiled the presence of subclinical Psoriatic Arthritis (PsA) in a substantial proportion, amounting to 16.45 %, of patients with moderate-to-severe Psoriasis (PsO) who were devoid of any articular symptoms. Pertaining to noteworthy imaging indicators, our findings underscore the importance of vigilant observation for enthesitis, synovitis, and new bone formation in the interphalangeal joint, knee joint, and tendon calcaneus among asymptomatic patients.

## Funding information

This work was sponsored by grants from 10.13039/501100001809National Natural Science Foundation of China (No. 82073429, 82273510, 82003334, 82001816 and 82072092), Innovation Program of 10.13039/501100003395Shanghai Municipal Education Commission (No.2019-01-07-00-07-E00046), Clinical Research Plan of SHDC (No. SHDC2020CR1014B) and Program of Shanghai Academic Research Leader (No. 20XD1403300).

## IRB approval status

Reviewed and approved by the Medical Ethical Committee of Shanghai Skin Disease Hospital (approval #2020-36).

## Data Availability Statement

All data used in the generation of the results presented in this manuscript will be made available upon reasonable request from the corresponding author.

## CRediT authorship contribution statement

**Rongfen Chen:** Writing – original draft, Data curation, Conceptualization. **Xiaoyuan Zhong:** Conceptualization. **Dawei Huang:** Conceptualization. **Zitong Chen:** Data curation. **Yingyuan Yu:** Data curation, Conceptualization. **Jiajing Lu:** Writing – review & editing. **Qiao Wang:** Investigation, Data curation. **Luyang Kong:** Investigation, Conceptualization. **Xuemei Yi:** Writing – review & editing. **Yujing Zhao:** Investigation. **Yangfeng Ding:** Writing – review & editing, Methodology. **Lehang Guo:** Writing – review & editing, Supervision, Methodology, Investigation, Funding acquisition. **Yuling Shi:** Writing – review & editing, Methodology, Investigation, Funding acquisition, Data curation.

## Declaration of competing interest

The authors declare that they have no known competing financial interests or personal relationships that could have appeared to influence the work reported in this paper.
